# Understanding the Information Needs and Context of Trauma Handoffs to Design Automated Sensing Clinical Documentation Technologies: Qualitative Mixed-Method Study of Military and Civilian Cases

**DOI:** 10.2196/17978

**Published:** 2020-09-25

**Authors:** Laurie Lovett Novak, Christopher L Simpson, Joseph Coco, Candace D McNaughton, Jesse M Ehrenfeld, Sean M Bloos, Daniel Fabbri

**Affiliations:** 1 Department of Biomedical Informatics Vanderbilt University Medical Center Nashville, TN United States; 2 Department of Emergency Medicine Vanderbilt University Medical Center Nashville, TN United States; 3 Geriatric Research Education and Clinical Center Tennessee Valley Healthcare System VA Medical Center Nashville, TN United States; 4 Department of Anesthesiology Vanderbilt University Medical Center Nashville, TN United States; 5 Department of Anesthesiology Medical College of Wisconsin Milwaukee, WI United States

**Keywords:** trauma handoffs, military field medicine, documentation, trauma, health records, hospital, emergency

## Abstract

**Background:**

Current methods of communication between the point of injury and receiving medical facilities rely on verbal communication, supported by brief notes and the memory of the field medic. This communication can be made more complete and reliable with technologies that automatically document the actions of field medics. However, designing state-of-the-art technology for military field personnel and civilian first responders is challenging due to the barriers researchers face in accessing the environment and understanding situated actions and cognitive models employed in the field.

**Objective:**

To identify design insights for an automated sensing clinical documentation (ASCD) system, we sought to understand what information is transferred in trauma cases between prehospital and hospital personnel, and what contextual factors influence the collection, management, and handover of information in trauma cases, in both military and civilian cases.

**Methods:**

Using a multi-method approach including video review and focus groups, we developed an understanding of the information needs of trauma handoffs and the context of field documentation to inform the design of an automated sensing documentation system that uses wearables, cameras, and environmental sensors to passively infer clinical activity and automatically produce documentation.

**Results:**

Comparing military and civilian trauma documentation and handoff, we found similarities in the types of data collected and the prioritization of information. We found that military environments involved many more contextual factors that have implications for design, such as the physical environment (eg, heat, lack of lighting, lack of power) and the potential for active combat and triage, creating additional complexity.

**Conclusions:**

An ineffectiveness of communication is evident in both the civilian and military worlds. We used multiple methods of inquiry to study the information needs of trauma care and handoff, and the context of medical work in the field. Our findings informed the design and evaluation of an automated documentation tool. The data illustrated the need for more accurate recordkeeping, specifically temporal aspects, during transportation, and characterized the environment in which field testing of the developed tool will take place. The employment of a systems perspective in this project produced design insights that our team would not have identified otherwise. These insights created exciting and interesting challenges for the technical team to resolve.

## Introduction

### Optimizing Communication Processes During Trauma Handoffs

When military personnel or civilians are injured, field medics are the first to respond. Their objectives include stabilizing and transporting the patient to a trauma facility. Optimizing patient outcomes depends on accurate information sharing between field personnel and receiving physicians, including the context of the injury and clinical interventions [1]. These patient and information transfers in combat settings are highly variable and can range from minimal communication (eg, pointing to a limb with a tourniquet when a helicopter picks up a patient from a scene in hostile territory) to verbal handoff when the patient is transported directly to the next level of care. When written documentation is generated, the documentation process may distract vital cognitive efforts away from patient care. Moreover, both written and verbal communication methods are vulnerable to rapid changes in clinical status, human cognitive biases, and mistakes in data collection, processing, and sharing. As a result, the information may be incomplete, inaccurate, or lost in communication [1-3]. Multiple handoffs further complicate the process and likely increase the risk of errors and miscommunication during transport.

Timely and accurate clinical documentation occurs when a sociotechnical system is designed and optimized around the relevant people, tasks, technologies, and physical and social environments [4]. Challenges include time pressure, the unique stress of providing care in combat situations, the use of personal protective equipment, limited visibility, and constrained working spaces. In addition, even when documentation is generated, it is rarely transmitted in a way that is timely, clear, or effective [5]. Given the challenges of using traditional technologies to document clinical care at the point of injury and during transport, new systems are needed that can ensure better, more consistent, and clearer communication among care teams.

Our main research effort is to develop novel technologies to automatically generate a clinical care record without requiring the active participation of personnel in the field [6]. This automated sensing clinical documentation (ASCD) technology observes the tasks the medic performs using a combination of sensors [7]. During its observation, the system outputs the list of clinical procedures that are being performed, ideally with high accuracy.

Designing ASCD involves understanding the other elements of the sociotechnical system into which the ASCD must fit [8]. These elements include information the system must capture and the social and physical contexts in which it will be deployed. Direct assessment of the current state of military trauma handoffs is impractical due to safety and logistical concerns [9]. Relying on civilian ambulance observations produces data from a limited number of trauma cases, typically in an environment that is unlike a military field operation. Therefore, through a multi-modal analysis that includes focus groups and trauma-bay video review, this paper analyzes current trauma handoff practices to categorize information needs and contextual factors involved in trauma handoffs.

### Background

The overall objective of our project is to develop an ASCD system that can be used on the battlefield by military personnel or by civilian medics in the field. The technology will involve a combination of off-the-shelf sensors, accelerometers, and cameras aligned with a software system that automatically detects the motion signatures associated with key clinical tasks and generates an abbreviated care record, which can be transmitted upstream in real time. The system will passively collect data from a combination of accelerometers and cameras. Machine learning, activity detection, and summarization algorithms will analyze the collected data to construct an abbreviated care record. This care record will provide patient clinical status, interventions, and anticipated resources needed upon arrival, without requiring active input from personnel in the field. Open research challenges to building such documentation systems include the accuracy of predicting clinical events, usability, and deployment robustness.

In the US conflicts in Iraq and Afghanistan, the nation has suffered a total death toll of 4432 and 2351 deaths, respectively, as of December 4, 2019 [10].^ ^ Since many fatalities occur between the point of injury and the medical treatment facility (MTF), the military has incorporated the use of Tactical Combat Casualty Care (TCCC) cards to document the mechanism of injury, injury locations, vital signs and symptoms, and treatments [11-13]. This allows the first responders to triage the most critical patients in the prehospital (eg, battlefield, vehicle) environment [12,13]. The military's documentation of the treatment during this period “is critical to ensuring continuity of care” [14]. After completing the card, the first responder attaches the TCCC card to the patient in a visible location as the record of provided treatment. Medical personnel in the receiving MTF are instructed to include the TCCC card with the paper medical record and to enter the TCCC data into the patient's electronic health record and appropriate trauma registry. Despite some evidence of a lack of compliance with the policy, the Defense Health Agency states,^ ^“The military will continue to use the TCCC card until it fields a prehospital documentation platform that supports an electronic version” [14].

The transfer of a patient from a field medic to an MTF is a handover, defined as “the transfer of professional responsibility and accountability for some or all aspects of care for a patient, or group of patients, to another person or professional group on a temporary or permanent basis” [15]. In this paper, we use the term “handoff” with the same definition. Handoffs in health care have received significant attention in recent years as a period of high risk for the patient's safety. In comparison with handovers in combat conditions, civilian handoffs are characterized by safe working conditions for the involved clinicians and an indoor Emergency Room physical setting with controlled lighting, climate, and (typically) noise. Additionally, trauma patients in civilian settings often undergo only one or two handoffs to reach the final hospital destination, whereas a patient in a combat situation may experience handoffs in several stages between the battlefield and the hospital. Despite these conditions, a review found that in civilian handoffs between medics and hospital-based emergency departments, the key issues were a lack of common understanding, lack of active listening, variable quality and quantity of information exchanged, lack of clear leadership, lack of teamwork skills, busy and complex environment, and handoff repetition [16]. Organizations have tried to resolve issues with handoffs through interventions to standardize communications, with mixed results [17,18]. Our project uses a systems perspective to examine an understudied topic that is especially challenging in military medicine: the capture of clinical documentation in the field, especially in battle conditions.

Findings in this paper are organized with a health care systems engineering model that has been extensively used in the study of both handoffs [19] and information technologies [20,21]. The Systems Engineering Initiative for Patient Safety (SEIPS) is a systems approach for understanding human activity in its context [22]. The fields of human factors and industrial engineering spurred the development of the framework to help frame research and innovation as technology was introduced into all areas of health care. The model was subsequently extended as SEIPS 2.0 to incorporate patient engagement, patient work, and work practice adaptations [23].

## Methods

### Research Questions

The research questions guiding this work were the following: (1) What information is transferred in trauma cases between prehospital and hospital personnel? (2) What contextual factors influence the collection, management, and handover of information in trauma cases?

### Research Site

Vanderbilt University Hospital provides trauma care for 65,000 square miles. The Division of Trauma at Vanderbilt University Hospital handles close to 5800 acute traumas, admitting 4300 of those annually. Its facilities are essential for the quality of trauma care provided by Vanderbilt University Hospital. These include a 20-bed burn unit and a 31-bed integrated acute and subacute care unit, which contains a 14-bed intensive care unit, a 7-bed acute admission area, and a 10-bed subacute unit, as well as LifeFlight, which is an active air medical transport program. The trauma units' unique geography allows close integration and management of patient progress from admission to discharge. LifeFlight provides rapid access to the tertiary care facilities of the Trauma Center for all patients within a 140-mile radius of Nashville. In addition to LifeFlight, Vanderbilt receives patient transport from local and rural emergency medical services (EMS).

### Research Approaches

#### Data Sources

Our methods included (1) a structured review of routinely captured videos of trauma handoffs in the Vanderbilt University Medical Center (VUMC) Emergency Department (ED), and (2) focus groups with ED providers, prehospital personnel (such as emergency medical technicians and paramedics), and military field medics. The research was conducted at VUMC and the Army's Rascon School of Combat Medicine on Fort Campbell, Kentucky.

The study protocols were reviewed and approved by the Vanderbilt University Institutional Review Board. Given the infeasibility of observational research of the activities of front-line military medical personnel, we used triangulation of data [24] from 2 different methods to gather information about the work of field medics (also referred to as prehospital personnel) and the handoffs between prehospital and hospital personnel.

#### Trauma Video Reviews

VUMC level 1 trauma cases are recorded for quality improvement purposes and reviewed weekly. These videos capture the pre-brief (in which EMS personnel and trauma team members from the ED and trauma team review facts about the arriving case and discuss a plan of action) and treatment while in the ED trauma bay. We reviewed 50 randomly selected videos to identify information transmitted via conversations during the handoff from EMS to hospital personnel. Videos are stored with no identifying linkages to patients and are deleted after a specified period. The videos were a way for us to observe handoffs without any patient-identifying information being collected.

A structured form facilitated the collection of relevant data from the videos. In order to refine a preliminary data collection form for the reviews, 5 videos were reviewed and documented by 3 reviewers. After the videos were reviewed, discussion of the results and any discrepancies in documentation were moderated by an independent arbiter. The reviewers came to a consensus on the types of information transferred from prehospital to hospital personnel and developed a data collection form to be used by 1 expert observer. The observer, a registered nurse, has extensive experience in trauma nursing and experience with the review of handoff videos. This observer viewed 50 trauma handoff videos and recorded observations on the forms. After completion of the reviews, the data from the observation forms were entered into a REDCap [25] database (Vanderbilt University) for analysis and tabulation.

#### Focus Groups

We conducted four 60-90-minute focus groups, each led by a team of 2 anthropologists (LN and CS) experienced in qualitative research. The focus group leaders had no prior relationship with the participants, although some participants had interacted with other research team members previously. The leaders had only general prior knowledge of the activities and contexts discussed in the focus groups and no personal experiences that would introduce bias.

Of the 4 focus groups that were conducted, 2 included civilian prehospital personnel (ambulance-based medics and aircraft-based flight medics), 1 included hospital personnel (physicians), and 1 was conducted with military combat medics. For the civilian focus groups, participants were recruited through email-based snowball sampling; for the military group, participants were recruited through a convenience sample of personnel available on the date of the study. Other members of the research team were present during the focus groups. There was no subsequent contact between the research team and the participants.

The goals of the focus groups were to gather information from providers and medics with trauma experience to (1) identify information transmitted in handoffs, (2) identify gaps in current handoff procedures, and (3) understand the social and physical context into which the technology will be deployed. These goals were communicated to the participants of each group. The sessions explored participant experience with the transportation of patients to the hospital, including the elicitation of actual experiences in a combat environment when possible. Questions posed during the focus groups included the following: (1) What information is normally shared during handoffs? (2) What information is most useful to determine the next steps of care management? (3) Why and how is this information shared? (4) What information is not useful to determine care management?

Based on the information shared in the session, we added probes to better understand the physical actions involved in transporting patients from the field or scene to the hospital, including the implications of incorporating wearable technologies, cameras, and other devices into the process.

The sessions were audio-recorded and transcribed for analysis. The transcripts were analyzed using a qualitative data analysis tool, Dedoose (SocioCultural Research Consultants), which facilitates the selection of text excerpts and labeling with one or more codes by a human coder and displays a variety of summaries of the coded data. Given the variety of information shared by participants on information needs and context, we used an open coding procedure, identifying all themes that arose in the data. There were 3 researchers who coded the data, supported by discussions in frequent team meetings about findings and the organization of the data. Then the data was organized using the SEIPS 2.0 model for presentation and consideration by the team's technology designers.

## Results

### Trauma Video Reviews

**Figure 1:**
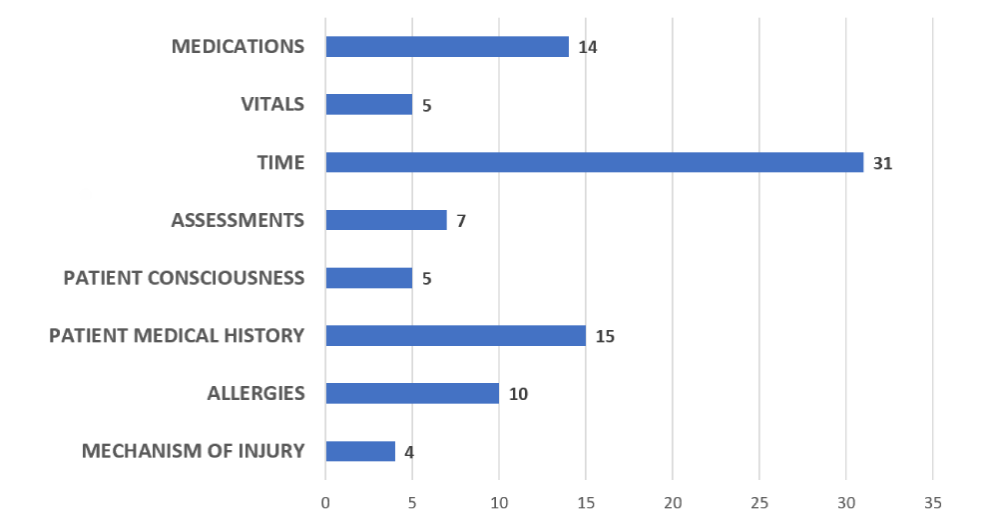
Content of clarifying questions in the trauma videos.

The handoff videos revealed information that is routinely relayed to the hospital team from the prehospital team. Figure 1 describes the content of each category of information in the 50 videos. Categories of information included clarifying questions asked by the receiving medical team, procedures performed, mechanism of injury, medications and fluids given during transport, time of intervention and injury, blood pressure, heart rate, respiratory rate, oxygen saturation, and episodes of hypotension changes in clinical status.

Upon analysis of the data, it became apparent that clarifying questions were an important part of the prehospital-to-trauma team handoff. Clarifying questions are defined as questions from the hospital team directed to the prehospital team during handoff that are intended to obtain additional information that was not provided in the initial handoff. Of the 50 videos reviewed, 40 (80%) contained clarifying questions.

The clarifying questions that we observed in the videos consisted of questions about medication (eg, dosages, timing), personal medical history (if known), Glasgow Coma Scale or other (mostly neurological) exam results, time and mechanism of injury, allergies, whether or not restraints were used in accidents in vehicles, length of time tourniquets have been in place, and fluctuations in vitals or neurological signs (blood pressure, heart rate, respiratory rate, oxygen saturation, etc).

**Figure 2:**
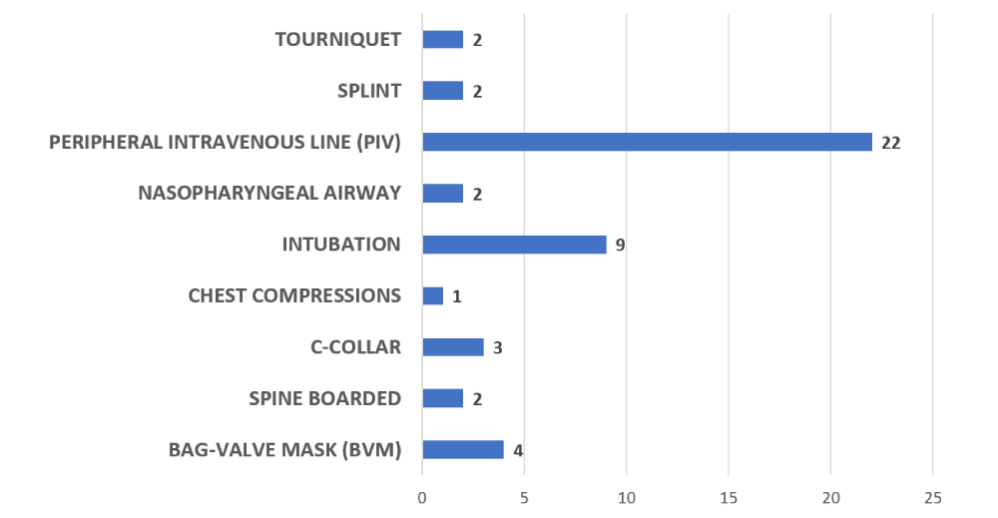
Frequency of procedures performed during transport in the trauma videos.

**Figure 3:**
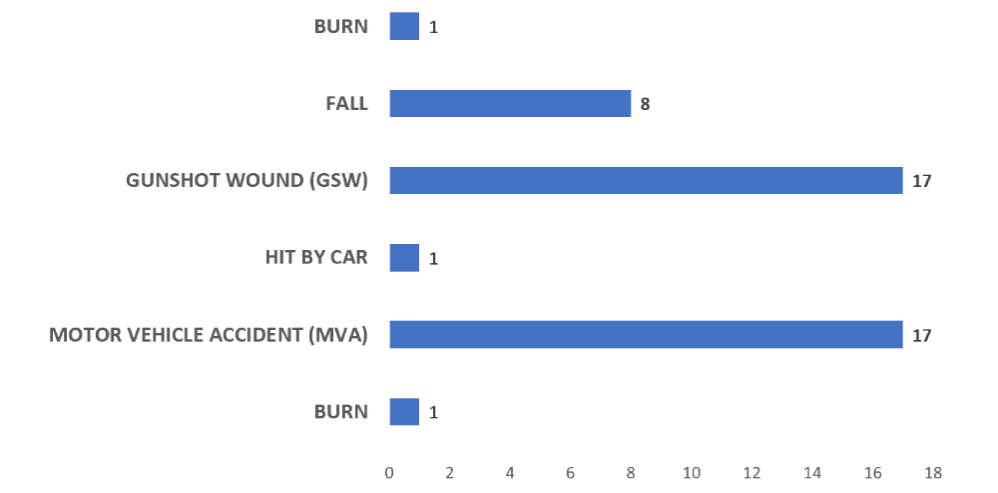
Frequency of each mechanism of injury in the trauma videos.

**Figure 4:**
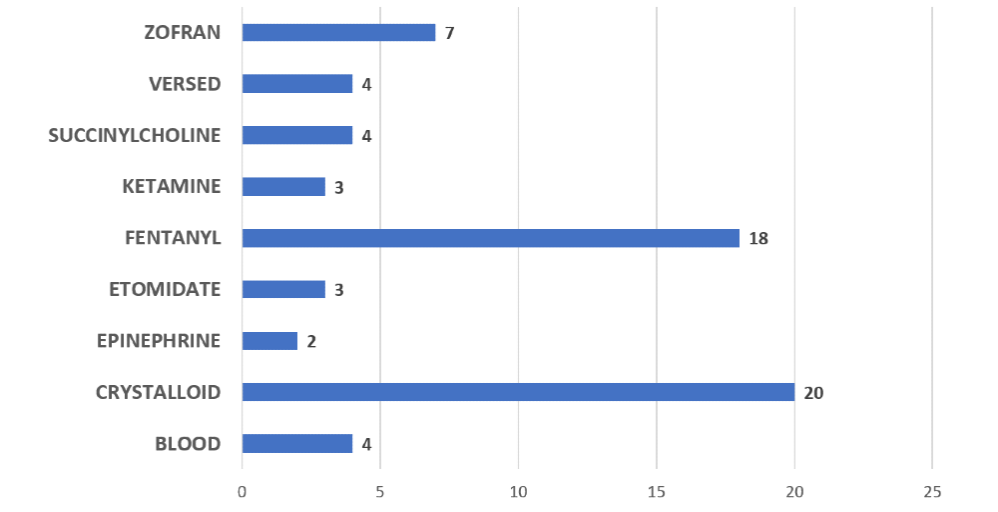
Medications and fluids administered during transport in the trauma videos.

**Figure 5:**
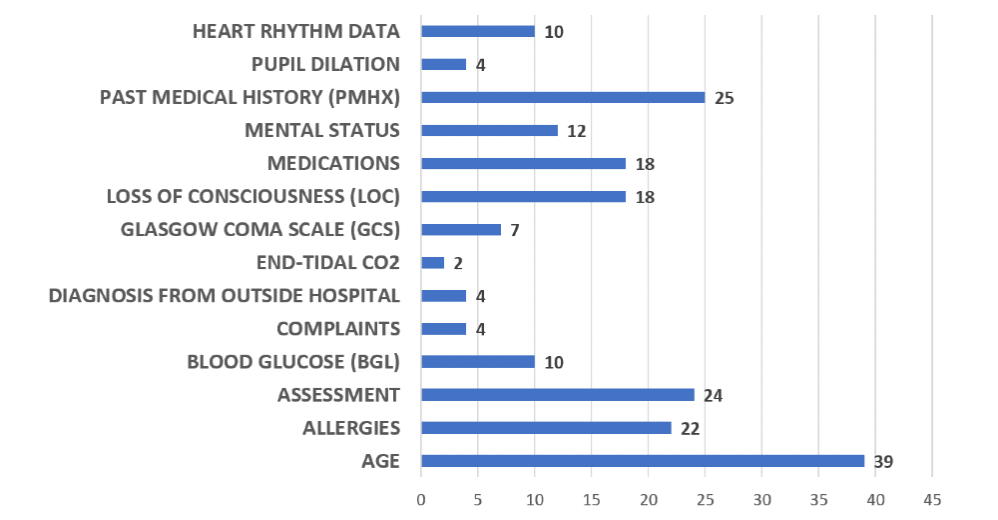
Other handoff information reported in the trauma videos.

The results for the other categories of information captured during observations are shown in Figures 2-5.

### Focus Groups

We conducted 4 focus groups comprising 19 participants, with no participants dropping out of the study. Participants included prehospital personnel (ambulance-based medics and aircraft-based flight medics), hospital personnel (physicians), and military medical personnel. Findings, comprising a comparison of the military and civilian experiences, are summarized using the SEIPS framework in Table 1.

**Table 1:** Work system analysis for documentation in the field.

Feature	Civilian prehospital system	Military prehospital system	Insights for the development of hardware and software tools
**Technology and tools**			
	Written or electronic documentation of prehospital care	TCCC^a^ (universal documentation card), sometimes partially completed by service member prior to mission	*Ad hoc use of tools, determined by the setting and characteristics of the patient *Information transmitted in advance could help hospital allocate resources (physician group) *In civilian setting, a simple statistic representing level of medic activity could potentially give early indication of severity of patient injury (physician group) *Mounting a camera in the vehicle is a challenge due to privacy issues in civilian setting *Object detection algorithms could potentially detect specific medication packages; however, packaging not currently standardized in military or civilian setting
	Gloves, paper, tape (used for recording written information)	Communication headsets	
	Vital signs monitoring technology	Medics carry medical gear and combat gear	
**Tasks**			
	Documentation: vital signs, demographics, medications, allergies, time of events, procedures, pain level, mechanism of injury	Documentation: vital signs, procedures, mechanism of injury	Priority information for handoff: *Timing and sequence of procedures can suggest cause and effect *Worst and most recent vital signs are most useful *In military setting, trends in data were more useful
	Procedures	Procedures	
	Radio communication	Radio communication	
	N/A^b^	Triage	
	N/A	Active combat activities	
	N/A	Maintaining tactical awareness	
**Organization**			
	Information systems in the EMS^c^ vehicle did not communicate with the hospital emergency department.	Large-scale, contracted military technology implementations sometimes lack coordination in technology updates, resulting in lost communication between system components.	Transmitting information to hospital can reduce miscommunication, but also result in information overload.
**Physical environment**			
	Extreme heat is common	Extreme heat is common, exacerbated by excessive gear	*Need lightweight, small sensors; armbands will be hot and uncomfortable *Voice technology not feasible because of noise *Sensors should conserve power when not in use *Wearable devices must withstand a substantial amount of sweat from the wearer *Devices must be physically durable
	N/A	Dusty	
	N/A	High noise level in all settings	
	N/A	Note-taking is difficult	
	N/A	Rough terrain/unstable vehicle	
	N/A	Low light in combat settings	

^a^TCCC: Tactical Combat Casualty Care.

^b^N/A: not applicable.

^c^EMS: emergency medical services.

## Discussion

### Principal Findings

Findings from the videos illustrated that the most medically important information is not always effectively conveyed during the handoff from prehospital to hospital personnel. Of note were the clarifying questions observed during the review of the videos of the handoffs. Clarifying questions were observed in 80% (40/50) of recorded handoffs and most commonly involved temporal aspects of the case. Temporal questions included queries about the time the injury occurred, when a procedure was performed, and when a medication was given. Questions related to timing (eg, when medications were administered) were present in 27 of the 40 videos in which clarifying questions were asked of the prehospital staff. The next most commonly asked clarifying question involved either medications (drugs given, doses, timing, etc) or the patient's past medical history. Both types of questions were present in 13 of the 40 videos in which clarifying questions were asked during the handoff.

Data from the observations support the findings from the 3 focus groups that more accurate information is needed at the time of handoff, specifically regarding time and sequences of procedures and medications. The hospital focus group emphasized that the most important information needed by the trauma team involved the timing of events, especially regarding the sequence of procedures performed during transport. The trauma videos revealed mechanisms of injury that would be less common in military environments (eg, falls and being hit by a motor vehicle). However, we note that it is difficult to speculate on what types of trauma injuries may be seen in future combat situations, and it is likely short-sighted to design only for wounds produced by gunshots or explosions.

The prehospital and hospital teams have different priorities and capabilities in the performance of their roles in their respective environments. Prehospital teams need to get the patient into the vehicle and perform needed procedures during transport to get the patient to superiorly resourced care teams, often geared toward surgical intervention. Meanwhile, the receiving trauma team wants to be able to appropriately allocate resources based on procedures performed and patient trajectory during transport. These differences result in an inadvertent conflict about the priority of recording specific times of medication administration and the performance and sequence of procedures during transport.

The findings from the video review and focus groups produced insights that informed device choices, software development, and evaluation strategy. Some surveillance technologies, such as microphones that could potentially be useful to support documentation, are not practical for noisy and insecure military field settings. While no tool will be able to capture every aspect of prehospital care, documentation through automated sensing can potentially enable medics to offer a more complete handoff to the receiving hospital.

### Implications for Design

Various activities are detectable through sensors. We identified numerous opportunities to capture activity (such as medical procedures or administration of medications) through motion detection and the relationship of motion signatures to locations on the patient's body, as well as the use of physical artifacts such as medication packaging. However, there is heterogeneity in how procedures are performed and noise in the data. A robust system of data collection and analysis will be needed to deal with the forces of real-world deployments. Challenges such as vehicle motion and sensor failure due to the environment (eg, a wearable sensor exposed to extensive sweat) may be universal. Challenges specific to military environments include the lack of lighting, a high possibility of network failure, and the possibility of active battle conditions while treatment is being carried out.

### Conclusion

An ineffectiveness of communication is evident in both the civilian and military worlds. We used multiple methods of inquiry to study the information needs of trauma care and handoff, and the context of medical work in the field. Our findings informed the design and evaluation of an automated documentation tool. The data illustrated the need for more accurate recordkeeping, specifically temporal aspects, during transportation, and characterized the environment in which field testing of the developed tool will take place. Solutions will need to address the environmental constraints of low lighting, heat, dust, noise, and vehicle instability. In addition, sensor power conservation is critical in field combat settings. The employment of a systems perspective in this project produced design insights that our team would not have identified otherwise. These insights created exciting and interesting challenges for the technical team to resolve.

## Abbreviations

ASCD: automated sensing clinical documentation
ED: Emergency Department
EMS: emergency medical services
MTF: medical treatment facility
SEIPS: Systems Engineering Initiative for Patient Safety
TCCC: Tactical Combat Casualty Care
VUMC: Vanderbilt University Medical Center
